# The risk of global Ebola virus spread is low: epidemiology of Ebola disease cases outside Africa, 1976 to May 2026

**DOI:** 10.2807/1560-7917.ES.2026.31.24.2600508

**Published:** 2026-06-18

**Authors:** Kevin van Zandvoort, Simon R Procter, James M Azam, Katharine Sherratt, Nicholas G Davies

**Affiliations:** 1 Centre for Mathematical Modelling of Infectious Diseases, London School of Hygiene and Tropical Medicine (LSHTM), London, United Kingdom; 2 Foreign, Commonwealth and Development Office (FCDO) Multi-Hazard Research Network, United Kingdom; 3 UK Public Health Rapid Support Team, United Kingdom

**Keywords:** Ebola virus transmission, Bundibugyo virus, international infectious disease transmission, travel restrictions, border screening, entry screening, Ebola disease

## Abstract

Following the Bundibugyo virus disease outbreak reported in the Democratic Republic of the Congo in May 2026, we reviewed all known Ebola disease cases outside Africa and found that intercontinental transmission risk remains low. We identified 28 confirmed epidemic-linked cases outside Africa; only four involved travellers with latent infection whose symptoms were detected after border screening. Excluding medically evacuated cases, the crude overall risk since 2000 was 0.17 Ebola disease cases outside Africa per 1,000 reported cases in Africa.

An outbreak of Ebola disease caused by Bundibugyo virus in the Democratic Republic of the Congo (DRC) was reported in May 2026, with additional cases subsequently detected in Uganda. Neighbouring countries are at greatest risk of cross-border spread, but decision-makers outside Africa may be considering border and travel policies to interrupt pathways for international transmission. We identified and analysed all known Ebola disease cases outside Africa to contextualise intercontinental spread and assess the risk of undetected *Orthoebolavirus* transmission outside Africa.

## Ebola disease cases outside Africa

We conducted internet searches for all laboratory-confirmed Ebola disease cases presenting outside Africa, performing both manual and artificial intelligence (AI)-assisted internet searches to identify scientific articles, public health bulletins and news reports without any time limit. Between 28 May and 8 June 2026, we used search terms such as “[country name] Ebola” to find sources and used snowball searching to locate additional reports. We manually extracted key epidemiological details for each case; AI-assisted searches were used only to identify cases and sources that manual searching may have missed, not to extract data. We checked our findings against existing lists of Ebola disease cases [1,2]. In total, we included 27 scientific articles, 9 reports in public health bulletins, 82 news articles and 9 other sites in gathering data. Full details of our search strategy are given in the Supplement. Full epidemiological details and sources are given in Supplementary Table 1.

We included all confirmed Ebola disease cases due to Bundibugyo, Ebola and Sudan virus outside Africa due to direct transmission from an ongoing outbreak in humans. This included both cases with exposure in Africa and subsequent travel outside the continent, as well as cases with exposure outside Africa. We did not include suspected cases with no confirmed test results. We excluded confirmed cases due to needle-stick injuries in laboratory workers handling infected material (one identified in the United Kingdom (UK), two identified in Russia), and a confirmed case of Taï Forest virus in a Swiss scientist exposed to an infected chimpanzee. We excluded animal-to-human Reston virus infections because Reston virus does not cause symptoms in humans [3].

**Figure 1:**
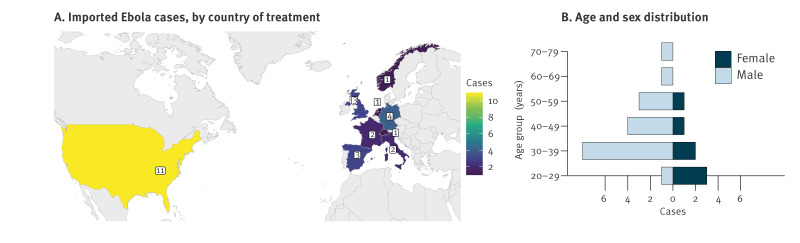
Confirmed cases of Ebola disease exported outside Africa, 1976–May 2026 (n = 28)

We identified 28 confirmed Ebola disease cases outside Africa: 25 primary imported cases and three secondary cases infected by another patient in the United States (US) or Europe (Table 1**,** Figure 1). Of these, 27 occurred during the 2014–16 Ebola virus epidemic in Western Africa and one has occurred so far during the ongoing 2026 Bundibugyo virus epidemic. No other historical Ebola disease outbreak led to exportation of cases from Africa. In our analysis, we distinguished between two types of primary exported cases: medically evacuated cases, who were securely transported by air ambulance for treatment outside Africa following a confirmed infection, and latent cases, who developed symptoms during or after their return from the outbreak region on a commercial flight.

**Table 1:** Summary of imported cases of Ebola disease outside Africa, 2014–2026 (n = 28)^a^

ID	Exposure in	Imported to	Occupation	Exposure	Age (years)	Outbreak responder	Medically evacuated	Died	Importation date	Source^b^
1	Liberia	France	Nurse	Nosocomial	Unclear	Yes	Yes	No	19 Sep 2014	N
2	Sierra Leone	France	UNICEF official	Community	Unclear	Yes	Yes	No	1 Nov 2014	N
3	Sierra Leone	Germany	Epidemiologist	Community	30–39	Yes	Yes	No	27 Aug 2014	[19]
4	Sierra Leone	Germany	Doctor	Nosocomial	30–39	Yes	Yes	No	3 Oct 2014	[20,21], N
5	Liberia	Germany	Laboratory technician	Laboratory	50–59	Yes	Yes	Yes	9 Oct 2014	[22], N
6	DRC	Germany	Doctor	Nosocomial	30–39	No	Yes	No	20 May 2026	B, N
7	Sierra Leone	Italy	Doctor	Nosocomial	50–59	Yes	Yes	No	25 Nov 2014	[23], N
8	Sierra Leone	Italy	Nurse	Nosocomial	30–39	Yes	No	No	9 May 2015	[10,11], N
9	Liberia	The Netherlands	UN peacekeeper	Community	Unclear	No	Yes	No	6 Dec 2014	[24], B, N
10	Sierra Leone	Norway	Doctor	Nosocomial	30–39	Yes	Yes	No	7 Oct 2014	N
11	Liberia	Spain	Missionary/hospital worker	Nosocomial	70–79	Yes	Yes	Yes	7 Aug 2014	N
12	Sierra Leone	Spain	Missionary/hospital worker	Nosocomial	60–69	Yes	Yes	Yes	22 Sep 2014	B, N
13	Spain^c^	Spain	Nurse	Nosocomial	40–49	No	No	No	NA	[25], N
14	Sierra Leone	Switzerland	Doctor	Nosocomial	40–49	Yes	Yes	No	21 Nov 2014	[26], N
15	Sierra Leone	UK	Nurse	Nosocomial	20–29	Yes	Yes	No	24 Aug 2014	[27], B, N
16	Sierra Leone	UK	Nurse	Nosocomial	30–39	Yes	No	No	28 Dec 2014	[8,28], B, N
17	Sierra Leone	UK	Military health worker	Nosocomial	20–30	Yes	Yes	No	12 Mar 2015	N
18	Liberia	US	Doctor	Nosocomial	30–39	Yes	Yes	No	2 Aug 2014	[29], N
19	Liberia	US	Nurse	Nosocomial	50–59	Yes	Yes	No	5 Aug 2014	[29], N
20	Liberia	US	Doctor	Nosocomial	50–59	Yes	Yes	No	5 Sep 2014	[30], N
21	Sierra Leone	US	Doctor	Nosocomial	40–49	Yes	Yes	No	9 Sep 2014	N
22	Liberia	US	Civilian	Community	40–49	No	No	Yes	20 Sep 2014	[31], N
23	US^c^	US	Nurse	Nosocomial	20–29	No	No	No	NA	[31], N
24	US^c^	US	Nurse	Nosocomial	20–29	No	No	No	NA	[31], N
25	Liberia	US	Photojournalist	Community	30–39	No	Yes	No	6 Oct 2014	N
26	Guinea	US	Doctor	Nosocomial	30–39	Yes	No	No	17 Oct 2014	[7], N
27	Sierra Leone	US	Doctor	Nosocomial	40–49	Yes	Yes	Yes	15 Nov 2014	N
28	Sierra Leone	US	Physician associate	Nosocomial	30–39	Yes	Yes	No	13 Mar 2015	N

B: public health bulletin(s); DRC: Democratic Republic of the Congo; ID: identification code; N: news article(s); NA: not applicable; UK: the United Kingdom; UN: the United Nations; UNICEF: the United Nations Children's Fund; US: the United States.

^a^ All cases were linked to the 2014–16 Ebola disease epidemic in Western Africa, except Case 6 which was linked to the 2026 Bundibugyo outbreak in DRC.

^b^ Public health bulletins and news articles are included in Supplementary Table 1.

^c^ Secondary transmission cases. Case 13 was infected while caring for Case 12; Cases 23 and 24 were infected while caring for Case 22.

No cases were reported in Asia, Latin America and the Caribbean, Oceania, or countries in Europe or North America. Therefore, they are not included in the above table.

Panel B. Three cases with unknown age and/or sex are omitted.

### Europe

In Europe, we identified 17 confirmed Ebola disease cases since 1976, including 16 primary cases and one secondary case in a healthcare worker treating a medically evacuated patient in Spain (Table 1). All 16 primary cases were workers with known occupational risk, including 12 healthcare workers, one United Nations (UN) peacekeeper, one laboratory technician, one epidemiologist and one United Nations Children's Fund (UNICEF) official. Of the 16 primary cases, 14 were medically evacuated due to confirmed Ebola virus infection, and two were latent cases, one a UK nurse who developed symptoms while returning to the UK, and one a Spanish healthcare worker who developed symptoms after returning to Spain. Of the 17 cases in Europe, 16 occurred during the 2014–16 Ebola disease epidemic in Western Africa and one during the 2026 Bundibugyo virus epidemic.

### North America

In North America, we identified 11 confirmed Ebola disease cases since 1976, including nine primary cases and two secondary cases in healthcare workers treating a patient in the US (Table 1). Of the nine primary cases, seven were frontline healthcare workers, one case was a photojournalist reporting on the epidemic, and one case was a traveller who did not have a responsive role in the epidemic. Of the same nine primary cases, seven were medically evacuated due to confirmed Ebola virus infection, while two were latent cases, one the aforementioned traveller and one a healthcare worker who developed symptoms after returning to the US. All 11 cases occurred during the 2014–16 Ebola disease epidemic in Western Africa.

We identified no confirmed Ebola disease cases in Latin America and the Caribbean, Asia or Oceania.

Overall, of the 28 confirmed Ebola disease cases, five died. All three secondary cases were due to nosocomial infection of healthcare workers treating a known Ebola disease patient.

## Low overall risk of exportation

As a crude measure of risk, we calculated for Ebola disease outbreaks since the year 2000 the number of cases occurring outside Africa per 1,000 reported cases in the originating epidemic. This gives a crude risk estimate of 0.81 cases outside Africa, including medically evacuated, latent and secondary cases, per 1,000 Ebola disease cases in a source outbreak (Table 2). Focusing on latent cases and their secondary cases only, there have been 0.17 cases outside Africa per 1,000 Ebola disease cases in the source outbreak.

**Table 2:** Crude risk calculation of cases infected with Ebola virus and exported outside the African continent by Ebola disease outbreak and overall, 2000–2026

Year	Country	Virus name	Cases (n)^a^	Medically evacuated cases (including onward transmission)	Crude risk: medically evacuated cases per 1,000 Ebola disease cases	Latent exported cases (including onward transmission)	Crude risk: latent exported cases per 1,000 Ebola disease cases	All exported cases (including onward transmission)	Crude risk: all exported cases (including onward transmission)
2000	Uganda	Sudan	425	0	0	0	0	0	0
2001	Congo^b^, Gabon	Ebola	124	0	0	0	0	0	0
2003	Congo^b^	Ebola	35	0	0	0	0	0	0
2003	Congo^b^	Ebola	143	0	0	0	0	0	0
2004	Sudan	Sudan	17	0	0	0	0	0	0
2005	Congo^b^	Ebola	12	0	0	0	0	0	0
2007	Uganda	Bundibugyo	131	0	0	0	0	0	0
2007	DRC	Ebola	264	0	0	0	0	0	0
2008	DRC	Ebola	32	0	0	0	0	0	0
2011	Uganda	Sudan	1	0	0	0	0	0	0
2012	Uganda	Sudan	6	0	0	0	0	0	0
2012	DRC	Bundibugyo	38	0	0	0	0	0	0
2012	Uganda	Sudan	11	0	0	0	0	0	0
2014	DRC	Ebola	69	0	0	0	0	0	0
2014	Sierra Leone, Liberia, Guinea, Nigeria, Mali	Ebola	28,646	21	0.73	6	0.21	27	0.94
2017	DRC	Ebola	8	0	0	0	0	0	0
2018	DRC	Ebola	3,470	0	0	0	0	0	0
2018	DRC	Ebola	54	0	0	0	0	0	0
2020	DRC	Ebola	130	0	0	0	0	0	0
2021	DRC	Ebola	11	0	0	0	0	0	0
2021	DRC	Ebola	12	0	0	0	0	0	0
2021	Guinea	Ebola	23	0	0	0	0	0	0
2022	Uganda	Sudan	164	0	0	0	0	0	0
2022	DRC	Ebola	1	0	0	0	0	0	0
2022	DRC	Ebola	5	0	0	0	0	0	0
2025	DRC	Ebola	64	0	0	0	0	0	0
2025	Uganda	Sudan	14	0	0	0	0	0	0
2026	DRC, Uganda	Bundibugyo	452^b^	1	2.6	0	0	1	0
All since 2000	Various	Various	34,362	22	0.64	6	0.17	28	0.81

CDC: Centers for Disease Control and Prevention; DRC: Democratic Republic of the Congo; INSP: Institut National de Santé Publique; US: the United States.

^a^ Historical number of reported cases from the US CDC [32] and elsewhere [33,34].

^b^ Congo (Brazzaville).

^c^ Ongoing outbreak; confirmed cases as of 5 June 2026, DRC INSP [35].

Of note, this represents the empirically observed risk of importation (and onward transmission) across all countries outside Africa; per-country risks are smaller. For example, focusing only on the UK, there has been one latent Ebola disease case since 2000, giving a risk estimate of 0.03 latent UK cases for every 1,000 Ebola disease cases in a source outbreak.

## Decrease in exportation risk over the 2014–16 epidemic

For cases exported from the 2014–16 epidemic in Western Africa, we carried out a time-varying risk analysis using Poisson regression of the number of exported cases for a given epidemiological week and country (across Guinea, Liberia and Sierra Leone, including weeks with no exported cases, from 6 January 2014 to 20 December 2015) against (i) the number of cases reported for that epidemiological week and country and (ii) the number of weeks since 6 January 2014 as a linear predictor of time-varying risk. We conducted this analysis using R version 4.5.1 (https://www.r-project.org/); see Supplement for details.

**Figure 2:**
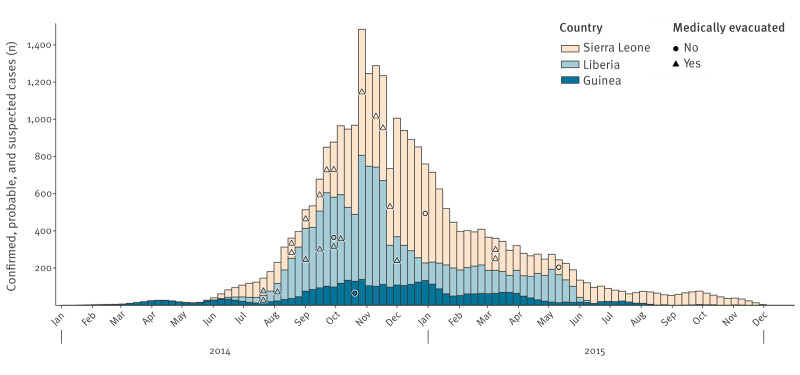
Timeline of importation cases of Ebola disease from the 2014–16 epidemic in Western Africa (n = 24)

The number of exported cases (both medically evacuated and latent) closely scaled with the number of cases in the source country in that week (Figure 2), with no significant deviation from linear scaling (estimated scaling: 0.98; 95% CI (confidence interval): 0.60–1.36; p = 0.86). Over the course of the epidemic, the case exportation risk per reported source outbreak case decreased by 4.6% per week (95% CI: 1.7–7.4%; p = 0.0097). More generally, the case exportation risk was substantially lower after the epidemic peak (0.46; 95% CI: 0.17–0.73 exported cases per 1,000 source cases from 27 October 2014 onwards) than it was before the peak (1.7; 95% CI: 0.95–2.7 exported cases per 1,000 source cases). This represents a 73% reduction in exportation risk per outbreak case between these two periods. As most exported primary cases during this epidemic (20 of 24) were medical evacuations, this is likely to be most consistent with improvement in infection prevention and control measures coinciding with the stepping-up of response efforts in late 2014, as further evidenced by decreasing transmission after the peak of the epidemic.

Weekly count of new confirmed, probable and suspected cases, by week and country for Guinea, Liberia and Sierra Leone (total 28,592). Overlaid points show the date of importation (x-axis), source country (colour) and medical evacuation status (triangles for medically evacuated cases, circles for latent cases) of primary exported cases (n = 24).

## Discussion

We identified 28 confirmed Ebola disease cases presenting in countries outside Africa. Agreement among search methods and with a clinical review of exported cases from 2014 to 2016 [2] support that our list is likely complete. Nonetheless, potential sources of under-ascertainment include that public health bulletins published not in English were underrepresented despite targeted native language searches, and web-based search tools may have returned geographically biased results.

A key distinction in our analysis is between medically evacuated and latent Ebola disease cases. While medically evacuated cases represent known risks that can be mitigated with strict biosecurity measures, latent cases arriving via commercial flights need to be diagnosed before they can be isolated. We identified four latent cases, all of which were exported during the 2014–16 Ebola disease epidemic. The earliest such case was a traveller from Liberia, who was exposed while helping their pregnant neighbour obtain medical assistance [4]. After returning home to the US, they presented to an emergency room with fever and were discharged, before eventually being readmitted and diagnosed with Ebola disease. This case prompted enhanced screening measures at five US airports designed to provide greater protection against similar cases [5,6].

The other three latent cases were returning healthcare workers responding to the epidemic. An American doctor developed fever, rapid breathing and fatigue several days after returning to the US from Guinea and immediately self-isolated and reported to health authorities for testing [7]. A British nurse developed fever and malaise on their return flight from Sierra Leone, was initially passed through screening at Heathrow airport after six normal temperature readings and notified local health services after their symptoms worsened at home [8]. An Italian nurse returned home from Sierra Leone, commenced self-monitoring of their temperature as per protocol [9], and immediately self-isolated and contacted health authorities upon developing fever, chills, muscle pain and weakness [10,11].

Notably, the four latent cases we identified occurred among 300,000 travellers screened by the US Centers for Disease Control and Prevention (CDC)-supported exit screening programmes in Guinea, Liberia and Sierra Leone, and all were asymptomatic (and hence undetectable) at the point of both exit screening [5] and entry screening [4,7-9]. Screening protocols included clear guidance on procedures for self-monitoring of symptoms [9], which ultimately led to rapid confirmation and isolation among the three latent cases in medical workers.

While our study focuses on cases presenting outside Africa, Ebola virus transmission to non-neighbouring countries within Africa is also historically rare. In the 2014–16 epidemic, only one of the affected African countries, Nigeria, did not share a land border with the others [12].

Previous work has evaluated interventions for mitigating international spread of Ebola virus. In model-based analyses of the 2014–16 epidemic, exit screening in affected countries was found to be more cost-effective than entry screening among incoming travellers [13]. However, any screening is limited due to non-specificity of early symptoms [5].

In 2026, in response to the ongoing outbreak, travel restrictions have been imposed for example, by Canada which has temporarily instituted a 21-day quarantine policy for travellers from DRC [14], and the US which has temporarily closed its borders to visitors and legal permanent residents who have recently visited DRC, South Sudan or Uganda [15]. While border closures plausibly reduce the immediate risk of importation [16], they can also hamper outbreak response and control in the source region [5].

Crucially, most Ebola disease cases reported outside Africa (27 of 28 cases) have been either (i) primary cases in individuals with a known responsive or occupational exposure to an Ebola disease epidemic (24 cases, including 20 healthcare workers, three UN employees and one journalist, among whom 20 were medically evacuated) or (ii) secondary cases in healthcare workers treating an Ebola disease patient outside Africa (3 cases). There was only one reported confirmed case outside Africa in a traveller with no responsive or occupational exposure. The one identified non-occupational case is a concern, especially as it led to two subsequent secondary cases. However, our results suggest overall that the risk of case exportations is low and could be substantially mitigated by infection prevention and control measures at the outbreak source and among outbreak response workers, in concert with enhanced travel screening and monitoring for returning response workers, as recommended in the World Health Organization (WHO) border and travel guidance for the current outbreak [17].

A proportionate approach with multiple options for managing travel-related risk may be the most appropriate response [5]. As exit screening in an outbreak-affected country aims to reduce case importations in other countries, it is a shared international responsibility. This may be best supported by strengthening local capacity for such screening [18].

## Conclusion

The risk of undetected Ebola virus transmission outside Africa is low. Nearly all confirmed Ebola disease cases reported outside Africa have been linked to known occupational exposures, with reasons for travel specific to outbreak response. We emphasise the role of local, community-based case management and infection prevention and control measures as the most effective strategy for managing outbreak risks both overall and outside the source region.

## Data Availability

Search results and analysis code may be found at https://github.com/cmmid/ebola-importation.

## Supplementary Data

259000 Supplementary Material
