# Supplementary material for: The risk of global Ebola virus spread is low: epidemiology of Ebola disease cases outside Africa, 1976 to May 2026
Source: Euro Surveill. 2026 Jun 18;31(24):2600508. doi: 10.2807/1560-7917.ES.2026.31.24.2600508 (PMC13282593; doi:10.2807/1560-7917.ES.2026.31.24.2600508)
Supplement: Supplementary Material [file 26-00508_DAVIES_Supplement.pdf]

Supplement to:

The risk of global Ebola virus spread is low: epidemiology of Ebola disease cases outside Africa, 1976 to May 2026

Kevin van Zandvoort, Simon R Procter, James Azam, Katharine Sherratt, Nicholas G Davies

This supplementary material is hosted by Eurosurveillance as supporting information alongside the article “The risk of global Ebola virus spread is low: epidemiology of Ebola disease cases outside Africa, 1976 to May 2026” on behalf of the authors, who remain responsible for the accuracy and appropriateness of the content. The same standards for ethics, copyright, attributions and permissions as for the article apply. Supplements are not edited by Eurosurveillance and the journal is not responsible for the maintenance of any links or email addresses provided therein.

Supplement

1. Search strategy

Eligibility criteria

The unit of analysis was confirmed human Ebola cases occurring outside Africa between 1976 and May 2026, restricted to the genus *Orthoebolavirus*; *Marburgvirus*, a related but distinct filovirus, was excluded throughout.

A case was eligible for inclusion if it was a laboratory-confirmed infection with Ebola (i.e. Zaïre) virus, Sudan virus, or Bundibugyo virus, presenting outside Africa through direct transmission from an ongoing human outbreak. In practice these comprised two routes: the medical evacuation of aid or health workers infected while serving in an active outbreak, or undetected (latent) cases in individuals who developed symptoms during or after their departure from an epidemic-affected country. For some importations, there was also subsequent human-to-human nosocomial transmission from imported cases to health care workers in the receiving country.

Four categories of event were excluded. First, suspected cases without a confirming laboratory result were excluded, including returned travellers who tested negative or were never confirmed, and health workers evacuated for monitoring after a high-risk exposure who did not seroconvert. Second, confirmed infections arising from needle-stick injuries in laboratory workers handling infected material were excluded; three such infections were identified, one in the United Kingdom (in Porton Down, 1976) and two in Russia (in Sergiev Posad, 1996 and in Koltsovo, 2004). The 1976 case was, additionally, the only Sudan virus infection identified outside Africa. A potential 2009 Hamburg laboratory needle-stick was likewise set aside, as the exposed worker received post-exposure vaccination and never developed confirmed disease. Third, the single confirmed human case of Taï Forest virus, in a Swiss scientist who autopsied an infected chimpanzee in 1994, was excluded. Fourth, animal-to-human infections with Reston virus were excluded because this species does not produce clinical illness in humans; these comprised handler seroconversions in the United States and two seroconversion events among animal and pig-industry workers in the Philippines.

Information sources and search strategy

We conducted internet searches for all confirmed Ebola cases presenting outside Africa, combining a manual search led by the authors with a complementary AI-assisted search. The manual search comprised internet searches conducted by the authors and cross-referenced against publicly available data on Ebola cases imported into Europe published by the European Centre for Disease Prevention and Control (ECDC) as well as a review of case management of exported cases from the 2014–16 Ebola outbreak in Western Africa (Uyeki *et al.* 2016). The AI-assisted search comprised internet searches for all Ebola cases outside Africa performed with two large-language models, Anthropic's Claude Opus 4.8 and OpenAI's GPT-5.2 Thinking model. The AI searchers used targeted web searches in both English and in the native language of each country focusing on one continent at a time. Both components covered every

region outside Africa, covering Europe, Northern America, Latin America and the Caribbean, Asia, and Oceania.

Sources were consulted in a fixed order of preference: peer-reviewed academic articles first; then public-health bulletins, for example from the World Health Organization, the ECDC, or national public-health bodies; and, where these were unavailable, news articles. Searching included non-English sources where feasible based on the languages of the authors, including in Dutch, French, and Italian. One eligible case, a confirmed Ebola virus patient medically evacuated to the Netherlands in December 2014, was identified only through a Dutch-language search, while other cases were initially identified through English-language sources.

Data collection

Searches were divided among all authors, and each author's results were independently double-checked by another author against the cited sources. For each identified case we collected: (i) age, sex, nationality, and name, the last used only for cross-referencing between sources; (ii) the source outbreak and country, the destination country, exposure details, frontline-worker status, medical-evacuation status, and key dates; and (iii) virological confirmation and clinical outcome (recovered or died). All data were extracted manually by the authors.

Study selection and verification

Candidate cases were screened against the eligibility criteria, and suspected cases without a confirmatory laboratory result were excluded. The results of the manual search were then checked against the AI-assisted search, which identified no additional cases meeting the inclusion criteria. The AI-assisted search therefore served only to detect cases that manual searching might have missed, and all case-level data were extracted manually by the authors.

2. Time-varying risk analysis

For cases exported from the 2014–2016 West African epidemic, we carried out a time-varying risk analysis using Poisson regression of the number of exported cases for a given epidemiological week and country (across Guinea, Liberia, and Sierra Leone, including weeks with zero exported cases, from 6 January 2014 to 20 December 2015) against (i) the number of cases reported for that epidemiological week and country and (ii) the number of weeks since 6th January 2014 as a linear predictor of time-varying risk.

To verify the robustness of the time-trend incidence, we verified residual autocorrelation in exported cases by inspection of residual autocorrelation function plots and by calculating Driscoll-Kraay panel heteroskedasticity and autocorrelation-consistent standard errors across 2–6 week bandwidths (Driscoll and Kraay 1998). For the number of epidemic cases by source country and epidemiological week, we used amalgamated data from WHO patient databases (Github “cmrivers/ebola” 2015) up to 23 August 2014, and data extracted from the WHO Ebola Response Roadmap Situation Report for cases from 24 August 2014 onwards (HDX 2016). We

applied a 4-week smoothing window to reported cases by week to adjust for clustered reporting artifacts.

We analysed the extracted data for the time-varying risk analysis and prepared the figures using R 4.5.1 and the packages data.table, readxl, sandwich, lmtest, and ggplot2.

References

Driscoll JC, Kraay AC (1998) Consistent covariance matrix estimation with spatially dependent panel data. *Rev Econ Stat.* 80: 549–60. http://dx.doi.org/10.1162/003465398557825

GitHub "cmrivers/ebola" (2015) Data for the 2014 global ebola Ebola outbreak in West Africa. In: GitHub; 15 Dec 2015 [Internet]. [cited 7 Jun 2026]. Available from: https://github.com/cmrivers/ebola

HDX (2016) Number of Ebola cases and deaths in affected countries. In: Humanitarian Data Exchange; 23 Mar 2016 [Internet]. [cited 7 Jun 2026]. Available from: https://data.humdata.org/dataset/ebola-cases-2014

Uyeki TM, Mehta AK, Davey RT Jr, Liddell AM, Wolf T, Vetter P, *et al*. (2016) Clinical management of Ebola virus disease in the United States and Europe. *N Engl J Med*. 374: 636-46. http://dx.doi.org/10.1056/NEJMoa1504874

Supplementary Table 1. Full extracted data, sources, and data dictionary

Case ID	Claude	OpenAI	Include	Complete	Imported to	Source outbreak	Exposure country	Exposure place	Primary	Year	Travel	Who?	Exposure	Nationality
1	24	6	Yes	Yes	France	2014-16 West Africa	Liberia	Paynesville	NA	2014	Medevac to Paris	Unnamed nurse (MSF)	Nosocomial	French
2	28	12	Yes	Yes	France	2014-16 West Africa	Sierra Leone	Unclear	NA	2014	Medevac to Paris	Unnamed official (UNICEF)	Community	Unclear
3	15	5	Yes	Yes	Germany	2014-16 West Africa	Sierra Leone	Unclear	NA	2014	Medevac to Hamburg	Unnamed epidemiologist (WHO)	Community	Senegal
4	16	10	Yes	Yes	Germany	2014-16 West Africa	Sierra Leone	Lakka	NA	2014	Medevac to Frankfurt-am-Meinz	Unnamed paediatrician	Nosocomial	Uganda
5	23	11	Yes	Yes	Germany	2014-16 West Africa	Liberia	Monrovia	NA	2014	Medevac to Leipzig	lab tech (UN)	Lab	Sudan
6	33	19	Yes	Yes	Germany	2026 DRC Bundibugyo	DRC	Bunia	NA	2026	Medevac to Berlin	doctor, medical missionary (Serge)	Nosocomial	US
7	27	14	Yes	Yes	Italy	2014-16 West Africa	Sierra Leone	Lakka	NA	2014	Medevac to Rome	doctor	Nosocomial	Italy
8	34	18	Yes	Yes	Italy	2014-16 West Africa	Sierra Leone	Goderich	NA	2015	Freetown - Casablanca - Fiumicino - Alghero	nurse	Nosocomial	Italy
9	NA	15	Yes	Yes	Netherlands	2014-16 West Africa	Liberia	Unclear	NA	2014	Medevac to Utrecht	Unnamed peacekeeper (UN)	Community	Nigeria
10	19	8	Yes	Yes	Norway	2014-16 West Africa	Sierra Leone	Bo	NA	2014	Medevac to Oslo	medical doctor (MSF)	Nosocomial	Norway
11	10	3	Yes	Yes	Spain	2014-16 West Africa	Liberia	Monrovia	NA	2014	Medevac to Madrid	missionary and hospital worker	Nosocomial	Spain
12	14	7	Yes	Yes	Spain	2014-16 West Africa	Sierra Leone	Lunsar	NA	2014	Medevac to Madrid	missionary and hospital worker	Nosocomial	Spain
13	18	9	Yes	Yes	Spain	Local transmission	Spain	Madrid	12	2014	NA	nurse	Nosocomial	Spain
14	26	13	Yes	Yes	Switzerland	2014-16 West Africa	Sierra Leone	Kerry Town	NA	2014	Medevac to Geneva	doctor	Nosocomial	Cuba
15	11	4	Yes	Yes	UK	2014-16 West Africa	Sierra Leone	Kenema	NA	2014	Medevac to London	nurse	Nosocomial	UK
16	30	16	Yes	Yes	UK	2014-16 West Africa	Sierra Leone	Kerry Town	NA	2014	Freetown - Casablanca - Heathrow - Glasgow	nurse and aid worker	Nosocomial	UK
17	31	17	Yes	Yes	UK	2014-16 West Africa	Sierra Leone	Kerry Town	NA	2015	Medevac to London	military health worker	Nosocomial	UK
18	8	20	Yes	Yes	US	2014-16 West Africa	Liberia	Paynesville	NA	2014	Medevac to Atlanta, GA	physician (Samaritan's Purse)	Nosocomial	US
19	9	21	Yes	Yes	US	2014-16 West Africa	Liberia	Paynesville	NA	2014	Medevac to Atlanta, GA	nurse and missionary worker (SIM USA)	Nosocomial	US
20	12	22	Yes	Yes	US	2014-16 West Africa	Liberia	Paynesville	NA	2014	Medevac to Omaha, NE	obstetrician and missionary worker (SIM USA)	Nosocomial	US
21	13	23	Yes	Yes	US	2014-16 West Africa	Sierra Leone	Kenema	NA	2014	Medevac to Atlanta, GA	physician (WHO)	Nosocomial	US
22	21	24	Yes	Yes	US	2014-16 West Africa	Liberia	Monrovia	NA	2014	Monrovia - Brussels - Dulles - Dallas Ft Worth	civilian	Community	Liberia
23	20	25	Yes	Yes	US	Local transmission	USA	Dallas, TX	22	2014	NA	nurse	Nosocomial	US
24	22	26	Yes	Yes	US	Local transmission	USA	Dallas, TX	22	2014	NA	nurse	Nosocomial	US
25	17	27	Yes	Yes	US	2014-16 West Africa	Liberia	Monrovia	NA	2014	Medevac to Omaha, NE	photojournalist (NBC)	Community	US
26	25	28	Yes	Yes	US	2014-16 West Africa	Guinea	Guéckédou	NA	2014	Conakry - Brussels - JFK	physician (MSF)	Nosocomial	US
27	29	29	Yes	Yes	US	2014-16 West Africa	Sierra Leone	Freetown	NA	2014	Medevac to Omaha, NE	surgeon	Nosocomial	Sierra Leone
28	32	30	Yes	Yes	US	2014-16 West Africa	Sierra Leone	Port Loko	NA	2015	Medevac to Bethesda, MD	physician associate (Partners in Health)	Nosocomial	US
**Excluded cases identified in initial searches in Europe and Northern America**														
101	1	1	No	Yes	UK	1976 DRC outbreak	UK	Porton Down	NA	1976	NA	lab tech (Porton Down)	Lab	UK
102	5	2	No	Yes	Switzerland	Animal exposure	Côte d'Ivoire	Taï National Park	NA	1994	Medevac to Basel	Unnamed ethologist	Dead chimpanzee	Switzerland
103	2	NA	No	No	6 animal handlers in Pennsylvania, seroconversion only									
104	3	NA	No	No	4 animal handlers in Texas, seroconversion only									
105	4	NA	No	No	Infection detected in macaques only, Italy									
106	6	NA	No	No	Infection detected in macaques only, USA									
107	7	NA	No	No	Germany 2009 needlestick, never confirmed									
108	35	NA	No	No	Russia 1996 needlestick									
109	36	NA	No	No	Russia 2004 needlestick									
**Further searches outside Europe and Northern America**														
201					Asia	6 Reston cases in Philippines, 2008; no confirmed human cases ever								
202					Latin Am. & Caribbean	No confirmed human cases ever								
203					Oceania	No confirmed human cases ever								

Supplementary Table 1, cont. Full extracted data, sources, and data dictionary

Case ID	Nationality	Age	Sex	Responder	Medevac	Where treated	Confirmed	Died	Death date	Exposure date	Onset date	Admission date	Diagnosis date	Confirmation date	Departure date	Arrival date	Discharge date
1	French	Unclear	Female	Yes	Yes	Bégin military hospital, Paris	Yes	No	NA	Unclear	16 Sept 2014	Unclear	17 Sept 2014	17 Sept 2014	19 Sept 2014	19 Sept 2014	4 Oct 2014
2	Unclear	Unclear	Unclear	Yes	Yes	Bégin military hospital, Paris	Yes	No	NA	Unclear	Unclear	Unclear	Unclear	Unclear	1 Nov 2014	1 Nov 2014	23 Nov 2014
3	Senegal	36	Male	Yes	Yes	UMC Hamburg-Eppendorf	Yes	No	NA	Unclear	18 Aug 2014	24 Aug 2014	23 Aug 2014	23 Aug 2014	27 Aug 2014	27 Aug 2014	26 Sep 2014
4	Uganda	38	Male	Yes	Yes	Frankfurt University Hospital	Yes	No	NA	Unclear	28 Sept 2014	28 Sept 2014	28 Sept 2014	28 Sept 2014	3 Oct 2014	3 Oct 2014	19 Nov 2014
5	Sudan	56	Male	Yes	Yes	Klinikum St Georg, Leipzig	Yes	Yes	14 Oct 2014	Unclear	Unclear	Unclear	6 Oct 2014	6 Oct 2014	8 Oct 2014	9 Oct 2014	NA
6	US	39	Male	No	Yes	Charité University Hospital, Berlin	Yes	No	NA	Unclear	Unclear	Unclear	18 May 2026	18 May 2026	Unclear	20 May 2026	6 Jun 2026
7	Italy	50	Male	Yes	Yes	INMI Lazzaro Spallanzani, Rome	Yes	No	NA	Unclear	20 Nov 2014	Unclear	24 Nov 2014	24 Nov 2014	24 Nov 2014	25 Nov 2014	2 Jan 2015
8	Italy	37	Male	Yes	No	INMI Lazzaro Spallanzani, Rome	Yes	No	NA	Unclear	10 May 2015	11 May 2015	12 May 2015	12 May 2015	7 May 2015	9 May 2015	10 Jun 2015
9	Nigeria	Unclear	Male	No	Yes	Calamiteitenhospitaal Utrecht UMC	Yes	No	NA	Unclear	Unclear	Unclear	3 Dec 2014	3 Dec 2014	Unclear	6 Dec 2014	22 Dec 2014
10	Norway	30	Female	Yes	Yes	Oslo University Hospital, Ullevål	Yes	No	NA	Unclear	4 Oct 2014	6 Oct 2014	5 Oct 2014	5 Oct 2014	6 Oct 2014	7 Oct 2014	20 Oct 2014
11	Spain	75	Male	Yes	Yes	Hospital Carlos III, Madrid	Yes	Yes	12 Aug 2014	Unclear	Unclear	1 Aug 2014	5 Aug 2014	5 Aug 2014	6 Aug 2014	7 Aug 2014	NA
12	Spain	69	Male	Yes	Yes	Hospital Carlos III, Madrid	Yes	Yes	25 Sep 2014	Unclear	Unclear	Unclear	20 Sept 2014	20 Sept 2014	21 Sep 2014	22 Sep 2014	NA
13	Spain	44	Female	No	No	Hospital Carlos III, Madrid	Yes	No	NA	Unclear	30 Sep 2014	6 Oct 2014	6 Oct 2014	6 Oct 2014	NA	NA	5 Nov 2014
14	Cuba	43	Male	Yes	Yes	Hôpitaux Universitaires de Genève	Yes	No	NA	Unclear	16 Nov 2014	17 Nov 2014	17 Nov 2014	17 Nov 2014	20 Nov 2014	21 Nov 2014	6 Dec 2014
15	UK	29	Male	Yes	Yes	Royal Free Hospital, London	Yes	No	NA	Unclear	22 Aug 2014	24 Aug 2014	22 Aug 2014	22 Aug 2014	24 Aug 2014	24 Aug 2014	3 Sep 2014
16	UK	39	Female	Yes	No	Royal Free Hospital, London	Yes	No	NA	25 Dec 2014	28 Dec 2014	29 Dec 2014	29 Dec 2014	29 Dec 2014	28 Dec 2014	28 Dec 2014	24 Jan 2015
17	UK	25	Female	Yes	Yes	Royal Free Hospital, London	Yes	No	NA	Unclear	Unclear	11 Mar 2015	11 Mar 2015	11 Mar 2015	12 Mar 2015	12 Mar 2015	27 Mar 2015
18	US	33	Male	Yes	Yes	Emory University Hospital, Atlanta	Yes	No	NA	Unclear	23 Jul 2014	Unclear	27 Jul 2014	27 Jul 2014	1 Aug 2014	2 Aug 2014	21 Aug 2014
19	US	59	Female	Yes	Yes	Emory University Hospital, Atlanta	Yes	No	NA	Unclear	22 Jul 2014	Unclear	26 Jul 2014	26 Jul 2014	4 Aug 2014	5 Aug 2014	19 Aug 2014
20	US	51	Male	Yes	Yes	Nebraska Medical Center, Omaha	Yes	No	NA	Unclear	29 Aug 2014	1 Sep 2014	1 Sep 2014	1 Sep 2014	4 Sep 2014	5 Sep 2014	25 Sep 2014
21	US	44	Male	Yes	Yes	Emory University Hospital, Atlanta	Yes	No	NA	Unclear	6 Sep 2014	6 Sep 2014	7 Sep 2014	7 Sep 2014	Unclear	9 Sep 2014	19 Oct 2014
22	Liberia	41	Male	No	No	Texas Health Presbyterian, Dallas	Yes	Yes	8 Oct 2014	15 Sept 2014	24 Sept 2014	28 Sept 2014	30 Sept 2014	30 Sept 2014	19 Sept 2014	20 Sept 2014	NA
23	US	26	Female	No	No	NIH Clinical Center, Bethesda	Yes	No	NA	Unclear	11 Oct 2014	11 Oct 2014	11 Oct 2014	11 Oct 2014	NA	NA	24 Oct 2014
24	US	29	Female	No	No	Emory University Hospital, Atlanta	Yes	No	NA	Unclear	14 Oct 2014	14 Oct 2014	15 Oct 2014	15 Oct 2014	NA	NA	28 Oct 2014
25	US	33	Male	No	Yes	Nebraska Medical Center, Omaha	Yes	No	NA	Unclear	1 Oct 2014	2 Oct 2014	2 Oct 2014	2 Oct 2014	5 Oct 2014	6 Oct 2014	22 Oct 2014
26	US	33	Male	Yes	No	Bellevue Hospital, NYC	Yes	No	NA	Unclear	23 Oct 2014	23 Oct 2014	23 Oct 2014	23 Oct 2014	14 Oct 2014	17 Oct 2014	11 Nov 2014
27	Sierra Leone (US resident)	44	Male	Yes	Yes	Nebraska Medical Center, Omaha	Yes	Yes	17 Nov 2014	Unclear	6 Nov 2014	15 Nov 2014	10 Nov 2014	10 Nov 2014	15 Nov 2014	15 Nov 2014	NA
28	US	34	Male	Yes	Yes	NIH Clinical Center, Bethesda	Yes	No	NA	Unclear	Unclear	Unclear	11 Mar 2015	11 Mar 2015	13 Mar 2015	13 Mar 2015	9 Apr 2015
**Excluded cases identified in initial searches in Europe and Northern America**																	
101	UK	38	Male	No	No	Coppetts Wood, London	Yes	No	NA	5 Nov 1976	11 Nov 1976	5 Nov 1976	11 Nov 1976	12 Nov 1976	NA	NA	25 Feb 1977
102	Switzerland	34	Female	No	Yes	University Hospital of Basel	Yes	No	NA	16 Nov 1994	24 Nov 1994	26 Nov 1994	Unclear	Unclear	30 Nov 1994	1 Dec 1994	8 Dec 1994
103																	
104																	
105																	
106																	
107																	
108																	
109																	
**Further searches outside Europe and Northern America**																	
201																	
202																	
203																	

Supplementary Table 1, cont. Full extracted data, sources, and data dictionary

Case ID	Onward transmission	Secondary cases	Scientific articles and case reports		Bulletins and news articles						Other sources	
1	No	0			News	News	News					
2	No	0			News	News						
3	No	0	Article	Abstract								
4	No	0	Article	Article	News							
5	No	0	Article		News	News						
6	TBC	TBC			Bulletin	News	News	News				
7	No	0	Article		News	News	News					
8	No	0	Article	Article	News	News	News	News	News	News		
9	No	0	Article		Bulletin	News	News	News			Information	
10	No	0			News	News						
11	No	0			News	News	News	News	News		Wikipedia	
12	Yes	1			Bulletin	Bulletin	News	News	News	News	Wikipedia	
13	No	0	Article		News	News	News					
14	No	0	Article		News	News	News					
15	No	0	Article		Bulletin	News	News				Wikipedia	
16	No	0	Article	Article	Bulletin	News	News	News			Wikipedia	Wikipedia
17	No	0			News	News	News	News	News		Wikipedia	
18	No	0	Article		News	News	News	News			Information	
19	No	0	Article		News	News	News					
20	No	0	Article		News	News	News	News				
21	No	0			News	News					Wikipedia	
22	Yes	2	Article		News						Wikipedia	
23	No	0	Article		News	News						
24	No	0	Article		News							
25	No	0			News	News	News	News				
26	No	0	Article		News	News	News					
27	No	0			News	News	News				Wikipedia	
28	No	0			News	News	News	News				
**Excluded cases identified in initial searches in Europe and Northern America**												
101	No	0	Article		News						Wikipedia	
102	No	0	Article									
103												
104												
105												
106												
107			Article									
108												
109												
**Further searches outside Europe and Northern America**												
201			Article	Article								
202			Article		Bulletin							
203					Bulletin	Bulletin						

Supplementary Table 1, cont. Full extracted data, sources, and data dictionary

**Case ID**	Case ID from manual searching
**Claude**	Case ID from AI search (Claude)
**OpenAI**	Case ID from AI search (ChatGPT)
**Include**	Yes if case meets inclusion criteria
**Complete**	Is record complete
**Imported to**	Country where case was treated
**Source outbreak**	Outbreak source of transmission
**Exposure country**	Country where exposure occurred
**Exposure place**	City / settlement where exposure occurred
**Primary**	If non-NA, case ID (manual) of infector
**Year**	Year occurred
**Travel**	Travel history of case
**Who?**	Occupation of case
**Exposure**	"nosocomial" indicates the case was infected while working in an Ebola treatment centre or hospital; "community" indicates community transmission; "lab" indicates transmission from infected material
**Nationality**	Nationality of case
**Age**	Age of case
**Sex**	Sex of case
**Responder**	Yes if case was responding to the epidemic as a health or aid worker
**Medevac**	Yes if case was medically evacuated
**Where treated**	Where case was treated in destination country
**Confirmed**	Yes for virological confirmation
**Died**	Yes if case died
**Death date**	Date of death if case died
**Exposure date**	Date of exposure if known
**Onset date**	Date of symptom onset if known
**Admission date**	Date of hospital admission if known. Note that this is the first inpatient admission, regardless of whether that occurred in the outbreak country or in the destination country. "Unclear" signifies it is not clear if the case was admitted to hospital before their evacuation
**Diagnosis date**	Date of clinical diagnosis (usually equal to confirmation date)
**Confirmation date**	Date of virological confirmation
**Departure date**	Date the case left the source country
**Arrival date**	Date the case arrived in the destination country
**Discharge date**	Date the case was discharged from hospital, if survived
**Onward transmission**	Yes if there was any onward transmission
**Secondary cases**	Number of secondary cases
**Sources**	Sources used to populate table. Article = scientific article, Bulletin = health or government bulletin, News = news article, Information = information page
